# Dr. Watson’s Introductory Lecture

**Published:** 1847-03

**Authors:** 


INTRODUCTORY LECTURE ON PRACTICAL EDUCATION IN MEDICINE. BY DR. WATSON, OF THE NEW YORK HOSPITAL.

The object of this lecture is to magnify the importance of hospital instruction, in comparison with lecture-room and dispensary teachings; and its author has certainly made out a strong case. According to his observation, many young gentlemen commence the practice of both physic and surgery with very little dexterity, for want of practical opportunities. In speaking of the hospital to which he belongs, he says:

"A couple of youths, fresh from their studies, were directed here, many years ago, to apply cupping-glasses to the temples of a negro woman. The cups were at first applied moderately heated; but not adhering, they were held over the lamp until nearly roasting hot, and then applied anew. Still they would not stick. The process was again and again repeated, all to no purpose. The poor woman roared, and the poor lads labored, until the superintendent of the hospital, attracted by the noise, at length relieved her from her tormentors—archly observing to them as he entered the ward, that 'they had better practice on each other until they had learned how.'

"Not long since, as is my frequent custom, I requested some of the young men accompanying me through the wards, to examine and report upon the nature of an inflammatory swelling. The first that came forward was, as I afterwards ascertained, one who had just passed the ordeal of his examination. He could detect nothing peculiar in the case. The second was a student of scarcely a year's standing, who had previously examined similar cases. He pronounced the swelling to be an abscess already in need of the lancet. I allowed him to apply this, and, to the no small mortification of the other, he gave exit to a large collection of purulent matter.

"The practice of our profession is made up of details like these, and a knowledge of these details, be assured, is never to be reached through lectures or deduced from abstract principles. The physician or surgeon, worthy of the name is such only so far as he is able to turn to advantage those innumerable and ever changing little circumstances of daily experience which language cannot teach. The senses of the student, as well as his intellectual faculties, require to be thoroughly and systematically indoctrinated: The study of principles may give employment to the mind, either in the closet or the class-room; but the hospital and the bedside are the schools for the senses. I had almost said, the only schools; there are others."

All this is true; but we must not forget that he whose knowledge is made up of practical details, is at last but an empiric. In the West and South, where students of medicine from the beginning are present (we cannot say always study) in the offices of their preceptors, it frequently happens, that they are better versed in the practical than the theoretical departments of the profession—can *do* many things which they cannot explain.

According to Dr. W., the hospitals of our eastern cities are very much neglected. But why are they? We fear that students are not so much to blame as the visitors, physicians and surgeons who manage those public charities. In the city of New York, there is one hospital, and five or six hundred students. Now, of what possible advantage would it be to these young gentlemen to crowd the wards of that establishment? They could neither see nor hear. Let an amphitheatre be erected contiguous to the wards, and a selection of patients daily brought into it, and there will be no lack of clinical students. As to serving in the hospital, of course but a small number could have the opportunity.

